# MRI to digital medicine diagnosis: integrating deep learning into clinical decision-making for lumbar degenerative diseases

**DOI:** 10.3389/fsurg.2024.1424716

**Published:** 2025-01-06

**Authors:** Baoyi Ke, Wenyu Ma, Junbo Xuan, Yinghao Liang, Liguang Zhou, Wenyong Jiang, Jing Lin, Guixiang Li

**Affiliations:** ^1^ Department of Spine and Osteopathy Surgery, Guilin People’s Hospital, Guilin, China; ^2^ Guangxi Key Lab of Multi-source Information Mining & Security, Guangxi Normal University, Guilin, China; ^3^ School of Artificial Intelligence, Naning Vocational and Technical University, Nanning, China; ^4^ Information Center, Wuxiang Hospital of Nanning Second People’s Hospital, Nanning, China; ^5^ Operation Room, Guilin People’s Hospital, Guilin, Guangxi, China; ^6^ Department of Traditional Chinese Medicine, Guilin People’s Hospital, Guilin, Guangxi, China

**Keywords:** MRI, deep learning, clinical decision-making, lumbar disc herniation, lumbar spondylolisthesis, digital medicine diagnosis

## Abstract

**Introduction:**

To develop an intelligent system based on artificial intelligence (AI) deep learning algorithms using deep learning tools, aiming to assist in the diagnosis of lumbar degenerative diseases by identifying lumbar spine magnetic resonance images (MRI) and improve the clinical efficiency of physicians.

**Methods:**

The PP-YOLOv2 algorithm, a deep learning technique, was used to design a deep learning program capable of automatically identifying the spinal diseases (lumbar disc herniation or lumbar spondylolisthesis) based on the lumbar spine MR images. A retrospective analysis was conducted on lumbar spine MR images of patients who visited our hospital from January 2017 to January 2022. The collected images were divided into a training set and a testing set. The training set images were used to establish and validate the deep learning program's algorithm. The testing set images were annotated, and the experimental results were recorded by three spinal specialists. The training set images were also validated using the deep learning program, and the experimental results were recorded. Finally, a comparison of the accuracy of the deep learning algorithm and that of spinal surgeons was performed to determine the clinical usability of deep learning technology based on the PP-YOLOv2 algorithm. A total of 654 patients were included in the final study, with 604 cases in the training set and 50 cases in the testing set.

**Results:**

The mean average precision (mAP) value of the deep learning algorithm reached 90.08% based on the PP-YOLOv2 algorithm. Through classification of the testing set, the deep learning algorithm showed higher sensitivity, specificity, and accuracy in diagnosing lumbar spine MR images compared to manual identification. Additionally, the testing time of the deep learning program was significantly shorter than that of manual identification, and the differences were statistically significant (*P* < 0.05).

**Conclusions:**

Deep learning technology based on the PP-YOLOv2 algorithm can be used to identify normal intervertebral discs, lumbar disc herniation, and lumbar spondylolisthesis from lumbar MRI images. Its diagnostic performance is significantly higher than that of most spinal surgeons and can be practically applied in clinical settings.

## 1 Introduction

Lumbar degenerative diseases encompass various conditions, such as lumbar spondylolisthesis, lumbar disc herniation, and lumbar spinal stenosis, which are summarized by the overall state of the lumbar spine. They represent significant causes of disability in the elderly population worldwide (1). Lumbar degenerative diseases are associated with a range of clinical symptoms, including persistent low back pain, varying degrees of leg pain, numbness, and difficulty walking, all of which can lead to a decrease in quality of life. Approximately 403 million people (5.5% of the global population) suffer from symptomatic intervertebral disc degeneration, and approximately 39 million people (0.53%) have spinal spondylolisthesis (2).

In recent years, the number of magnetic resonance imaging (MR imaging or MRI) examinations has significantly increased. According to the recommendations of the American College of Radiology, lumbar MRI can be used to exclude the aetiology of complex low back pain and determine whether conservative treatment or surgical intervention should be considered (3, 4). MRI is a noninvasive technique that provides cross-sectional and sagittal images for defining compressed nerve roots and vertebral slippage. Regarding the radiological diagnosis of lumbar degenerative diseases, MRI is the most appropriate noninvasive adjunctive imaging modality (5).

Artificial intelligence (AI) has demonstrated immense potential for transforming healthcare and medical imaging, with deep learning being one of the most impactful AI tools (6, 7). Increasingly, researchers have applied machine learning techniques to study various spinal conditions, including degenerative spine disorders (8, 9). AI deep learning technology enables the identification and corresponding diagnosis of multiple structural lesions, including intervertebral discs, vertebral bodies, and ligaments. The application of AI deep learning technology in disease diagnosis not only reduces the time required but also reduces the risk of missed or erroneous diagnoses. Currently, there is considerable research on the diagnosis and treatment of lumbar disc herniation using AI deep learning technology, but the quality varies, and accurate identification of multiple degenerative spine diseases remains challenging. In light of the common occurrence of lumbar disc herniation and lumbar spondylolisthesis in spinal surgery, this study aims to develop an intelligent system based on AI deep learning algorithms that can identify lumbar MR images to assist in the diagnosis of these two prevalent spinal conditions. Furthermore, the clinical usability of this system is validated to enhance the diagnostic and therapeutic efficiency of clinical physicians.

## 2 Materials and methods

A retrospective analysis was conducted on the general data of patients with lumbar degenerative diseases who visited Guilin People's Hospital from January 2017 to January 2022. The patients' lumbar spine MR images and clinical diagnostic reports were collected using a picture archiving and communication system (PACS). The inclusion criteria were as follows: (1) patients who visited our hospital with symptoms such as low back pain, radiating leg pain, numbness in the legs, or difficulty walking and underwent lumbar MRI examination; (2) complete lumbar MR images diagnosed by radiologists as lumbar spondylolisthesis and lumbar disc herniation; (3) no history of previous lumbar spine surgery; (4) no spinal tumours or metabolic bone diseases; and (5) no significant history of trauma. The exclusion criteria were as follows: (1) incomplete lumbar MR images or images with motion artefacts that made identification difficult; (2) history of previous lumbar spine surgery; (3) personal history of spinal tumours or metabolic bone diseases; and (4) significant history of trauma. The selected cases were randomly divided into two groups according to the ratio of 8:2: a testing group and a training group. The testing group was used to establish and validate the deep learning algorithm, while the training group was used to verify the accuracy of the model and conduct preliminary clinical validation.

### 2.1 Image preprocessing and annotation

**Figure 1:**
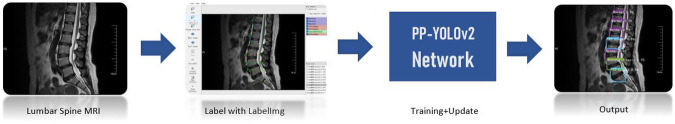
Illustration of the image annotation and training process.

Using a PACS system, lumbar spine MRI images of all patients were extracted from the DICOM database and saved as JPEG files with a resolution of 812 × 662 pixels. In this study, three spinal surgeons with different levels of experience manually annotated the regions of interest on the JPEG images using computer software called LabelImg (10). LabelImg is a deep learning annotation tool developed in Python that provides a user-friendly interface and supports various deep learning frameworks, such as Pascal VOC, YOLO, and TensorFlow annotation formats. It improves annotation efficiency and quality (11). During the annotation process, the surgeons adjusted the size and position of the bounding boxes using LabelImg to annotate different structures in each lumbar spine MRI image. The annotations were saved in XML file format for training the deep learning models. The surgeons referenced sagittal, axial, and T1/T2 images, as well as the patient's lumbar spine x-rays (including anteroposterior, lateral, and dynamic views) and lumbar spine 3D computerized tomography (CT) scans to determine lumbar spinal slippage. They then identified and annotated the normal intervertebral discs, lumbar disc herniation, and lumbar spinal slippage. The annotation task was independently performed by three experienced and specialized spinal surgeons (two of whom were individually annoted and were confirmed by the other, the most senior physician, when inconsistency occurred), in order to ensure the accuracy and reliability of the annotations. The annotation and training workflow is illustrated in Figure 1.

### 2.2 Establishment and validation of the deep learning algorithm

To develop a target-based detection algorithm for spinal disease diagnosis, this study utilized a deep neural network-based object recognition and localization algorithm, specifically the You Only Look Once (YOLO) series algorithms (12). The YOLOv3 (13) and YOLOv5 (14) algorithms are representative algorithms in the YOLO series. The PP-YOLO network model (15) is a novel object detector based on the Baidu deep learning framework PaddlePaddle. PP-YOLO was developed based on the optimization strategy of YOLOv3, with the ResNet50vd-DCN model with deformable convolution as one of the main optimization strategies. The PP-YOLOv2 model (16) captures information about small-scale targets using the Mish activation function and achieves high performance. So, we had used the PP-YOLOv2 model by the transfer deep learning method to develop the software of spinal disease diagnosis (17). The PP-YOLOv2 model showed the highest accuracy in diagnosing normal, disc herniation, and spinal slippage cases, with an overall accuracy of 90.08%. The diagnostic time was much faster than manual diagnosis by spinal surgeons, averaging 2 min (18). To show the performance of the different deep learning models, the spinal disease MRI dataset was trained on three target detection models: YOLOv3, YOLOv5, and PP-YOLOv2. We adopted the 5-fold cross-validation technique to get the result of different deep learning models. The mean average precision (mAP) values of the auxiliary diagnostic results obtained by the three models were 70.64%, 86.66%, and 90.08%, respectively (as shown in Table 1).

**Table 1:** Experimental results of different network models.

Deep learning model	Normal AP (%)	IVD bulges AP (%)	Spondylolisthesis AP (%)	mAP (%)	Speed (s)
YOLOv3	81.17	69.06	61.68	70.64	16.2
YOLOv5	91.77	85.43	82.77	86.66	**13** **.** **7**
PP-YOLOv2	**93** **.** **84**	**91** **.** **74**	**84** **.** **67**	**90** **.** **08**	14.5

Bold values indicate the performance of every column evaluation indicator was the best.

### 2.3 Accuracy validation and initial clinical validation of the model

Fifty lumbar spine MRI images in JPEG format were independently evaluated and annotated by both the deep learning algorithm and three spinal surgeons. The surgeons were allowed to use software functionalities to zoom in on the images and adjust image contrast. The first surgeon was a junior spinal surgeon with 4 years of experience in the field. The second surgeon was a mid-level spinal surgeon with 8 years of experience, and the third surgeon was a senior spinal surgeon with 15 **y**ears of experience. Additionally, three experts participated in the final evaluation of the testing group images. They included an MRI specialist with 25 years of experience in radiology, a spinal surgery expert with 30 years of experience, and another spinal surgery expert with 25 years of experience. To ensure the accuracy and reliability of the evaluations, the three experts independently assessed the 50 testing group images, accounting for the clinical histories, x-rays, and CT scans of each patient to enhance reliability. In cases of disagreement, discussions were held to reach a consensus. After determining the standardized evaluation answers, these answers were used to verify the diagnostic accuracy of the deep learning algorithm and the spinal surgeons.

Each doctor independently diagnosed an MRI image, recording the symptoms of each disc and summarizing all diagnoses. The training datasets include the annotation with 2,532 for normal, 1,467 for IVD bulges, and 585 for spondylolisthesis. The experimental environment for this article is Ubuntun16.04, which is based on Baidu's deep learning framework PaddlePaddle2.0, and Python 3.6. The GPU is an Nvidia Tesla V100 with 32GB of memory.

### 2.4 Statistical analysis

Statistical analysis of the data was performed using IBM SPSS 26.0 software. Sensitivity, specificity, accuracy, and time taken for the AI intelligent analysis software and surgeons with different levels of experience to diagnose normal intervertebral discs, disc herniation, and lumbar spinal slippage were analysed. Chi-square tests were performed on all data, with a significance level of *P* < 0.05 indicating a statistically significant difference.

## 3 Result

### 3.1 Dataset analysis

In the final results, general data from a total of 654 patients were included in the study. Among them, 604 cases were randomly selected as the training group for model establishment and recognition, while the remaining 50 cases were used as the testing group to compare and verify the accuracy of the final model's recognition results. The general data of the 654 patients are presented in Table 2.

**Table 2:** General data of included patients.

General information		Training group (604)	Test group (50)
Gender (M/F)		31%/69% (187/417)	36%/64% (18/32)
Average age		62.71	61.62
Slip site	L1	2.22% (13)	4.29% (3)
	L2	4.44% (26)	11.43% (8)
	L3	11.80% (69)	8.57% (6)
	L4	41.88% (245)	38.57% (27)
	L5	39.65% (232)	37.14% (26)
Lumbar disc herniation^a^	L1–2	12.88% (189)	14.63% (18)
	L2–3	17.18% (252)	19.51% (24)
	L3–4	21.06% (309)	22.76% (28)
	L4–5	15.34% (225)	11.38% (14)
	L5-S1	19.63% (288)	13.01% (16)
	Total lumbar spine	1,260 (86.09%)	100 (81.30%)
Thoracic disc herniation^a^	T9–10	0.34% (5)	0% (0)
	T10–11	2.52% (37)	3.25% (4)
	T11–12	5.39% (79)	8.13% (10)
	T12-L1	5.66% (83)	7.32% (9)
	Total thoracic spine	204 (13.91%)	23 (18.70%)

^a^ As most lumbar spine MR examinations include the lower thoracic segment, these data were annotated and recognized in both the training and testing groups of this study.

### 3.2 Accuracy of the deep learning algorithm

**Figure 2:**
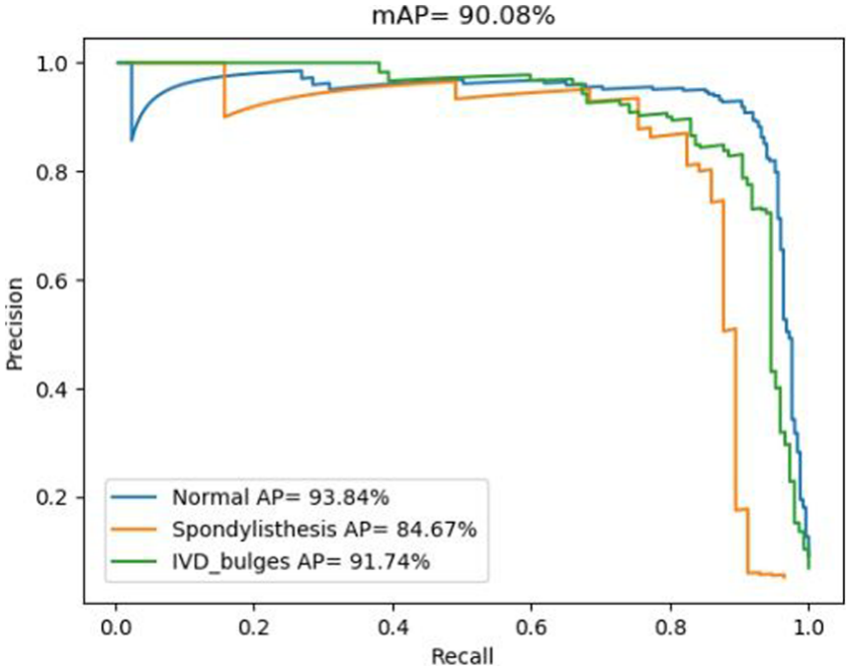
P-R diagnostic curves based on the transfer learning PP-YOLOv2 model.

In fact, the P-R curve graph is generated according to the training datasets during the training process of the deep learning model. The training datasets and the internal test datasets often are independent. In this study, in order to illustrate the better performance based on the spinal MRI Images based on the transfer learning PP-YOLOv2 model, the deep learning algorithm achieved an mAP value of 90.08% for the multiclass object detection task involving normal, herniated, and slipped discs (Figure 2).

The accuracy for each class is represented by the area under the corresponding precision-recall (P-R) curve, with a larger area indicating higher accuracy. The blue P-R curve represents a diagnostic accuracy of 93.84% for normal intervertebral discs, while the cyan P-R curve represents a diagnostic accuracy of 91.74% for disc herniation. The orange P-R curve represents a diagnostic accuracy of 84.67% for spinal slippage, which is lower due to the lower prevalence of slippage cases in actual clinical practice compared to normal intervertebral discs and disc herniation. The lower accuracy for spinal slippage is also attributed to the smaller size of the dataset available for training.

### 3.3 Comparison of accuracy of the deep learning algorithm and spinal surgeons

**Figure 3:**
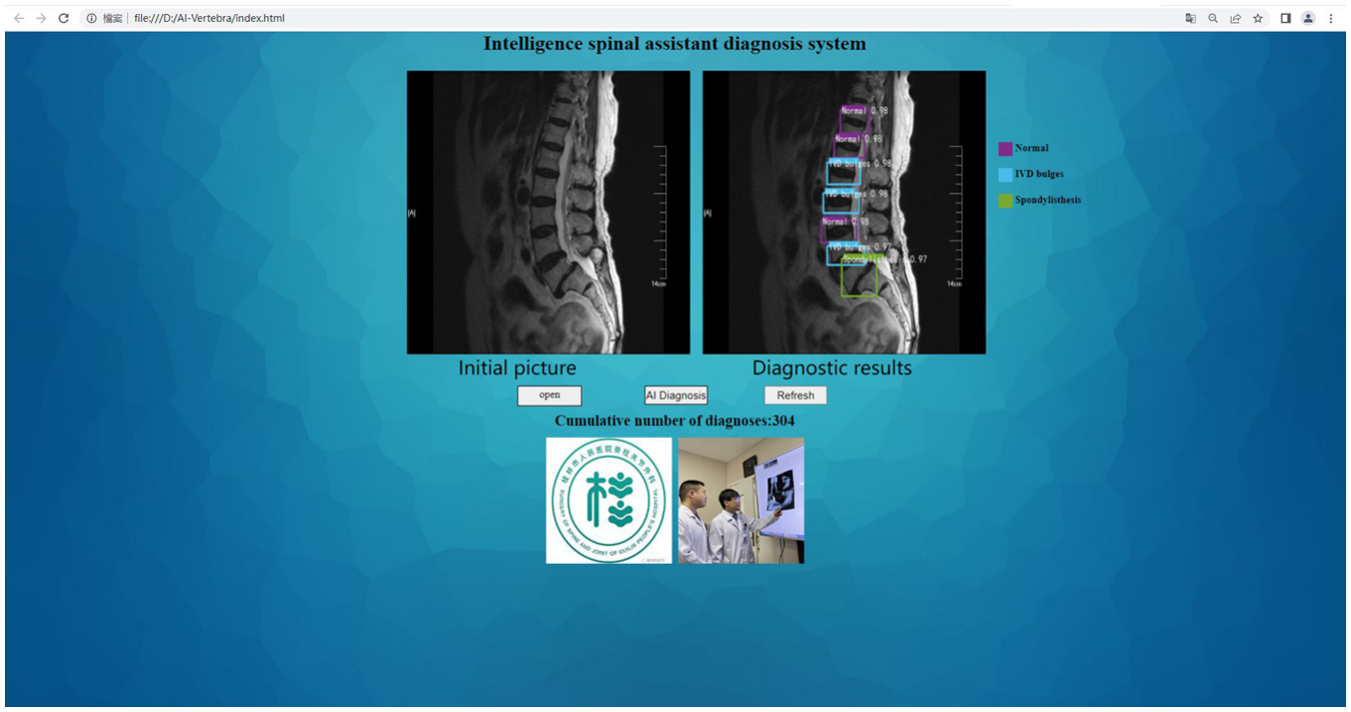
Example of lumbar spine MR image recognition by the deep learning algorithm.

Using the established deep learning algorithm, the training group of 50 data samples was validated, and the results were recorded (Figure 3).

Subsequently, the three spinal surgeons independently read and annotated the same 50 data samples from the training group, and their recorded data were analysed in conjunction with the standard results obtained by the three experts. The comprehensive statistical analysis was then performed as presented in Table 3. Additionally, the deep learning model exhibited significantly shorter diagnostic times than the surgeons (*P* < 0.05).

**Table 3:** Comparison of the diagnostic performance of the three spinal surgeons and the deep learning algorithm.

Doctor	Sensitivity	Specificity	Accuracy	Average time (s)
Physician 1	74.75% (148/198)	93.78% (181/193)	84.14% (329/391)	87
Physician 2	76.77% (152/198)	92.75% (179/193)	84.65% (331/391)	89.3
Physician 3	90.91% (180/198)	94.30% (182/193)	92.58% (362/391)	92.5
AI system	**97.98%** (194/198)	**98.45%** (190/193)	**98.21%** (384/391)	**14.5**

Bold values indicate the performance of every column evaluation indicator was the best.

**Figure 4:**
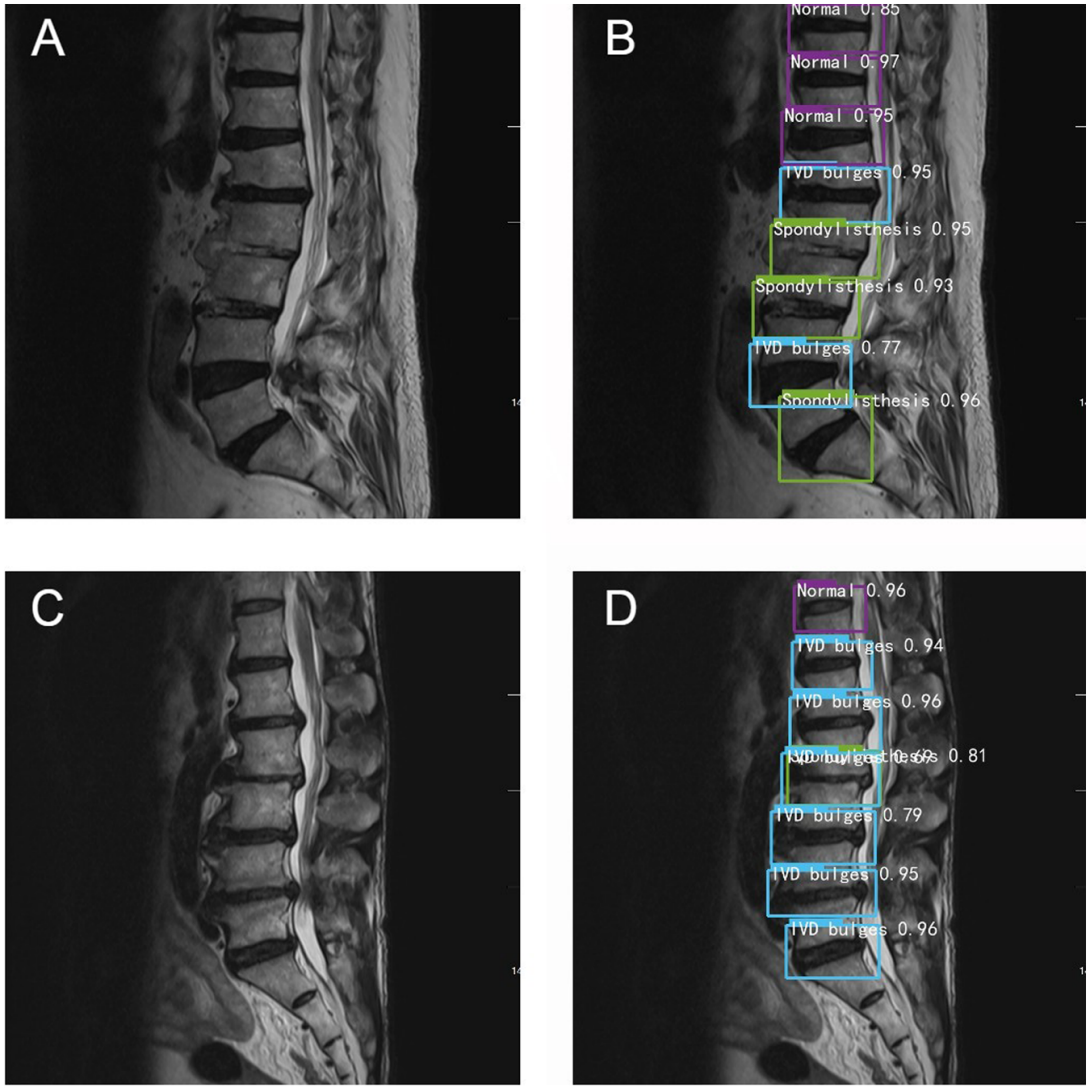
Typical schematic diagram of manual recognition **(A,C)** and deep learning model recognition **(B,D)**.

Data analysis revealed that with increasing years of experience, the sensitivity, specificity, and accuracy of spinal surgeons in diagnosing lumbar spine MR images im-proved. However, there was still a gap between their diagnostic performance and that of the deep learning model (Figure 4).

Among the collected cases, there are two cases in which the accuracy of deep learning model recognition is significantly higher than that of manual recognition. For example, in Figure 4A, the surgeons only identify spondylolisthesis of L5, but in the same image, the deep learning model also identifies spondylolisthesis of L2 and L3 in Figure 4B. In Figure 4C, the doctors judge that L2-L3 is a simple disc herniation, while in Figure 4D, the deep learning algorithm not only identifies L2 spondylolisthesis but also considers that there is disc herniation. Overall, the diagnostic accuracy of the deep learning algorithm was significantly higher than that of the three spine surgeons.

## 4 Discussion

Overall, the diagnostic mAP of the deep transfer learning algorithm reached 90.08%. In the diagnostic test of lumbar spine MRI for 50 patients, the algorithm achieved a diagnostic accuracy of 98.21%, surpassing that of the three spine surgeons. In terms of diagnostic efficiency, the algorithm's average diagnostic speed of 14.5 s was 6 times faster than the average speed of manual diagnosis. Clearly, the performance of the deep transfer learning algorithm is significantly superior to that of most spine surgeons.

Although most deep learning studies on lumbar spine MRI tend to focus on distinguishing the lower thoracic and lumbar vertebrae (19), some studies have achieved high accuracy in specific tasks. For example, Zhou et al. (20) combined a convolutional neural network (CNN) and preannotated lumbar spine images to identify lumbar verte-brae in MRI images of 1,318 healthy and unhealthy subjects, achieving an accuracy of 98.6%. They found that most identification failures were related to incorrect positioning of the first sacral vertebra or missed identification of the fifth lumbar vertebra. Tsai et al. (21) manually labelled lumbar disc herniation and lumbar vertebrae positions and trained a YOLOv3 CNN model to detect lumbar disc herniation and localize lumbar vertebrae in MRI images of 168 subjects, achieving an accuracy of 81.1% after data augmentation. Lehnen et al. (22) trained a CNN that can segment intervertebral discs and detect disc protrusion, compression, bulging, spinal stenosis, nerve root compression, and spinal instability, with 100% accuracy for vertebra detection and segment labelling, 86.8% accuracy for disc protrusion, and 87.6% accuracy for spinal instability. However, for the YOLO algorithm, which only requires identifying problem areas once, the artificial distinction between the thoracic and lumbar vertebrae appears unnecessary. Therefore, in this study, we annotated both the standard lumbar intervertebral discs and the lower thoracic vertebrae of the MR images.

We designed this algorithm and software to be used by medical professionals; the distinction between lumbar and lower thoracic vertebrae is common knowledge for medical professionals, including medical students. As a medical diagnostic assistance algorithm, we only require the algorithm to identify “problem areas,” while the specific segment localization is left to the judgement of the physician. The benefits of this approach are as follows: first, it reduces the time and manpower required for annotation, thereby improving the training efficiency of the deep learning algorithm; second, if segmentation is performed on the lumbar vertebrae, it may result in poor segmentation results or even errors in cases with edge blurring or morphological variations in the MR images. Not performing segmentation increases the robustness of the deep learning algorithm and enhances its recognition and analysis capabilities for different morphologies of lumbar spine images. Third, it simplifies the algorithm and reduces the risk of errors. By not requiring the algorithm to differentiate between the lower thoracic and lumbar segments, we reduce the complexity of the algorithm and minimize the possibility of errors. This is particularly important in the medical field, where errors can have serious consequences. Collaboration between healthcare professionals and algorithms can lead to more accurate diagnoses and better patient outcomes. De et al. (23) discussed the application of deep learning algorithms in the diagnosis and referral of retinal diseases. By training and testing on 14,893 retinal images from 9,962 participants, the researchers found that deep learning algorithms can be used to achieve highly accurate diagnoses and referral decisions for various retinal diseases. Furthermore, they found that combining deep learning algorithms with the clinical experience of doctors can further improve diagnostic accuracy. In summary, not artificially labelling and distinguishing between the lower thoracic and lumbar vertebrae can simplify the annotation process to some extent, improve efficiency, enhance practicality, and provide better scalability, making the application of deep learning algorithms in lumbar spine MRI recognition more widespread and reliable.

In current clinical practice, MRI is considered the imaging standard for diagnosing lumbar disc herniation. For lumbar spondylolisthesis, MRI is increasingly recognized as the best diagnostic method, complemented by lumbar spine x-rays and CT scans, to obtain a more definitive diagnosis. In deep learning, establishing the fundamental facts is a key step to ensure the performance and reliability of the model. The fundamental fact of this study is that MRI is already the primary method and standard for spine surgery di-agnosis. The goal of the deep learning algorithm is to automatically extract rich feature information from MR images and make judgements based on it. This study has the po-tential to impact doctors and patients in clinical settings. Importantly, with the use of our deep learning algorithm, the misdiagnosis and underdiagnosis of disc herniation and spondylolisthesis are expected to decrease. This is because the algorithm not only indicates the presence or absence of relevant lesions but also highlights suspicious lesions on the MR images, which will benefit the patients. Additionally, using this algorithm will enhance patient safety by minimizing radiation exposure, as the algorithm effectively identifies lumbar spondylolisthesis using MRI alone. Furthermore, our algorithm can assist not only spine specialists but also primary care physicians, emergency physicians, and other specialists who may encounter lumbar spine MR images in their daily clinical practice.

The strength of this model lies in its ability to rapidly diagnose disc herniation and spinal spondylolisthesis. Analysing the training data for normal discs, disc herniation, and lumbar spondylolisthesis, we find the accuracy for identifying normal discs is as high as 93.84%, reaching 91.74% for disc herniation, while the accuracy drops to 84.67% for lumbar spondylolisthesis. Although we have employed transfer learning and data augmentation techniques to improve accuracy, the limited number of actual clinical samples and the substantial workload involved in annotating samples lead to a decrease in accuracy. In our future work, we will expand the sample size and improve the quality of annotations to further enhance the training quality.

Although our algorithm achieves high diagnostic accuracy, it only answers the question of “presence or absence.” It does not address the question of “how severe” the disc herniation and spondylolisthesis subtypes are or provide more precise quantitative measurements. This idea will be a focus in our future work. AI cannot surpass human capabilities, as the training data labelling and establishment of fundamental facts for creating algorithms must be done by humans. Currently, our deep transfer learning model can only provide auxiliary diagnosis for normal discs, disc herniation, and lumbar spondylolisthesis. We also excluded spinal fractures, tumours, and scoliosis when selecting samples, so the diagnostic scope remains limited. Moreover, the sample collection was restricted to lumbar spine MR images. Purely radiological studies cannot fully integrate patient symptoms and signs to provide more accurate clinical treatment recommendations. Therefore, one of our future research directions will be to expand the sample range and external validation spinal datasets from different cooperative hospitals, including spinal fractures, tumours, spinal stenosis, and scoliosis, and broaden the research scope to include the cervical and thoracic spine. We will also explore the use of other medical imaging techniques (CT scans, x-rays, etc.) for sample collection and model training to further expand the applicability of the model. Additionally, there is still insufficient research on cross-sectional MR images. We will attempt to test the diagnosis software based on deep learning methods in prospective study or clinical trials, which maybe have some barriers, for example, data privacy is hard to come by and data from different hospitals are heterogeneous. We also will attempt to combine sagittal and transverse plane images for exploration and expect to discover more interesting findings.

## 5 Conclusions

In this study, we developed a deep learning algorithm based on the PP-YOLOv2 algorithm, which can identify normal intervertebral discs, disc herniation, and lumbar spondylolisthesis on lumbar spine MRI images and annotate them. The AI algorithm achieved higher diagnostic accuracy and faster diagnostic time than most specialized spine surgeons in the tests. We developed user-friendly software based on this algorithm, which has been applied in clinical practice and medical education with positive feedback. We believe that our algorithm, using different diagnostic criteria than humans, can significantly improve the accuracy of diagnosing disc herniation and spondylolisthesis when using lumbar spine MRI images.

## Data Availability

The original contributions presented in the study are included in the article/Supplementary Material, further inquiries can be directed to the corresponding author.
